# Enhanced PTC Effect in Polyamide/Carbon Black Composites

**DOI:** 10.3390/ma15155400

**Published:** 2022-08-05

**Authors:** Julian Nagel, Thomas Hanemann, Bastian E. Rapp, Guido Finnah

**Affiliations:** 1 Robert Bosch GmbH, Powertrain Solutions, Engineering Tank Unit and Innovation, Wernerstraße 51, 70469 Stuttgart, Germany; 2 Department of Microsystems Engineering (IMTEK), University of Freiburg, Georges-Köhler-Allee 102, 79110 Freiburg im Breisgau, Germany or; 3 Karlsruher Institut für Technologie, Institut für Angewandte Materialien (IAM), Herrmann-von-Helmholtz Platz 1, 76344 Eggenstein-Leopoldshafen, Germany; 4 Laboratory of Process Technology, NeptunLab, Department of Microsystems Engineering (IMTEK), University of Freiburg, 79110 Freiburg im Breisgau, Germany; 5 Freiburg Materials Research Center (FMF), University of Freiburg, 79104 Freiburg im Breisgau, Germany; 6 FIT Freiburg Center of Interactive Materials and Bioinspired Technologies, University of Freiburg, 79110 Freiburg im Breisgau, Germany

**Keywords:** nanocomposites, electrical properties, thermal properties, injection molding

## Abstract

Self-heating nanocomposites with a positive temperature coefficient (PTC) provide outstanding potential for a broad range of engineering applications in automobile, spacecraft, or smart building. Therefore, extensive studies have been carried out to understand thermo-electrical behavior. However, some controversies remain, especially on the material composition, to clarify influencing factors on the PTC performance. In this study, the thermo-electrical behaviors of injection molded carbon black (CB)/polyamide (PA) nanocomposites have been investigated. Three types of CB with well-defined specific surface area and polyamides with high and low crystallinity were selected to provide a guideline for self-heating devices including PTC-Effects. Significantly reduced specific resistances up to 2.7 Ω·cm were achieved by incorporating CB with a high specific surface area into a highly crystalline PA. Noticeable PTC-Effects of ~53% and average surface temperatures up to 147 °C have been observed due to self-heating, which confirms a promising material performance as a heating device.

## 1. Introduction

With the rapid development of functional and intelligent devices, there is an increasing demand for new materials, which can respond to external stimuli like strain [1] humidity [2], damage [3], or temperature [4]. Combining different polymers with conductive fillers, electrical, mechanical, or chemical properties can be tailored and tuned for the desired requirements. Such conductive polymer composites (CPCs) show a specific insulating/conductive [5] state transition and provide a large variety in their material composition, which can be easily fabricated. 

**Figure 1:**
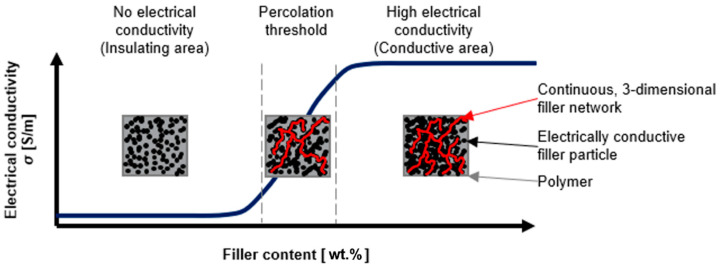
Schematic representation of the electrically conductive filler network within the non-conductive polymer [17,18].

To receive the electrical conductivity of non-conductive materials, such as polymers, researchers have focused on incorporating several types of fillers such as metallic [6,7,8], graphite [9,10], carbon nanotubes [11,12], or carbon black [13,14]. The state of the continuous, three-dimensional filler network within the polymer and, therefore, the electrical properties of the composites, strongly depends on the filler amount [6,12], aspect ratio [15], state of dispersion [12,16], and more (Figure 1).

In summary, composites with high amounts of well-dispersed fillers with high aspect ratios will reach the percolation threshold earlier and provide lower room temperature resistivities. This is attributed to the increasing number of filler-to-filler contacts and therefore a higher number of conductive paths. 

Certain composites show temperature-sensitive changes in their electrical resistivity, known as positive temperature coefficient (PTC) or negative temperature coefficient (NTC), where the resistivity increases or decreases, respectively, with increasing temperature [13]. 

Different theories have been proposed for the origin of PTC-Effects, but all somehow address the mismatches of thermal expansion of the materials used and the number of filler-to-filler contacts. Thereby, the electrically conductive filler particles within the polymer matrix get separated from each other during the thermal expansion of the polymer matrix. Due to this disturbance of the filler-to-filler connectivity, the electrical resistance increases. Research has revealed that this disturbance of the filler network, during the thermal expansion of the polymer matrix, is more distinct at low filler content [5,6,19], fillers with low aspect ratio [20], and increasing filler particle size [6]. These factors can be attributed to the lower number of contact points between the filler particles, which can be described as a less robust filler network. Therefore, the disturbance vulnerability of the filler network is higher, which results in higher PTC intensities. 

The occurrence of the NTC-Effect has not yet been fully understood but reaggregation effects of the filler particles, above the melting point of the polymer, are suggested. In the molten state of the polymer, the filler particles are forming new filler paths accomplished due to the higher filler mobility, which results in decreased electrical resistance [21]. 

Re-aggregation of the filler particles and, therefore, the occurrence of the NTC-Effect, can be eliminated due to crosslinking of the polymer, which supports the assumption of the NTC occurrence [22]. Further effects for the NTC behavior are suggested.

These properties, especially the PTC behavior, make these composites proper for sensor technical applications [23,24] or self-regulating heaters [19]. Such self-regulating heaters based on polymers provide excellent advantages over conventional inorganic PTC materials, such as light weight, formability, and flexibility. Furthermore, they do not reach too-high temperatures during self-heating caused by PTC behavior. For that reason, no complicated control units are necessary to limit the temperature rise. 

The use of composites as heating elements offers a wide range of potential applications, such as large area deicing components, thermo-clothing, or interior heating systems for the automotive sector. 

Despite this application potential, most of the research trends are primarily focused on the achievable electrical resistance at room temperature or the electrical resistance of the composites during storage at different temperatures. 

Less is known about the explicit use of conductive composites as self-heating devices at different applied voltages. Likewise, there have been no reports on the achievable self-heating and self-regulating performance of the polyamide composites investigated in this study. 

For this reason, the present work clarifies the PTC-Heater properties, as a function of the polymer crystallinity and specific surface area of incorporated carbon black to manufacture high self-heating nanocomposites, with superior PTC behaviors. 

In the current study, injection-molded polyamide (PA)/carbon black (CB) nanocomposites have been manufactured. Appropriate methods to reduce the percolation threshold and to enhance the self-heating performance of the composites significantly have been achieved due to highly crystalline polyamide and CB with high specific surface area. Furthermore, composites exhibit tunable PTC-Effects. 

## 2. Materials and Methods

### 2.1. Materials

Two variants of Polyamide (PA), PA 6.10 (BASF SE, Ludwigshafen, Germany) with a degree of crystallinity of 30–40% [25] and PA 4.6 (DSM Engineering Materials B.V., Emmen, The Netherlands) with a crystallinity of 60–70% [25] were used as received. Three types of carbon black (CB), CB I, and CB II (Imerys Graphite & Carbon, Bironico, Switzerland) with a specific surface area of 70 m^2^/g and 770 m^2^/g, as well as CB III (Orion Engineered Carbons GmbH, Frankfurt, Germany) with a specific surface area of 1000 m^2^/g, were used without any specific treatment or purification. For electrical contacting of the specimens, a silver-filled epoxy (Loctite Ablestik 84-1LMI, Henkel AG & Co. KGaA, Düsseldorf, Germany) was used.

#### Melt Compounding

The melt compounding process was carried out on a twin-screw extruder (ZSK 26 Mc Megacompounder) with a screw length-to-diameter ratio of 44, which was endowed with the Feed Enhancement Technology (FET) from Coperion GmbH, Stuttgart, Germany. The polymer strands spilled out of the compounder and went through a water bath of 2 m length, followed by a strand pelletizer (Type SP50 Pure) from Coperion GmbH, Stuttgart, Germany. A screw speed of 600 rpm and throughput of 20 kg/h was kept constant for every composite. Composites with CB contents of 10 wt.%, 15 wt.%, 20 wt.%, and 25 wt.% were produced.

### 2.2. Methods

#### Injection Molding

Before the injection molding, the samples were dried in a vacuum oven (Thermo VACUtherm VT 6060M, Fisher Scientific GmbH, Schwerte, Germany) for 4 h at 100 °C and 150 mbar. Afterward, plates with dimensions of 80 × 80 × 2 mm^3^ were injection molded (E-motion 220 T, Engel Austria GmbH, Schwertberg, Austria) with a screw diameter of 25 mm and a screw position depending on the switchover. The injection molding parameters are listed in Table 1 and Table 2. The holding pressure (15 s) as well as the dosage volumes of PA 6.10 composites (32 cm^3^) and PA 4.6 composites (34 cm^3^) were kept constant. 

### 2.3. General Characterization

A Belsop-mini X (Microtrac Retsch GmbH, Haan, Germany) was used to verify the specific surface area (BET) of CB according to DIN 6613. The microstructure of the composites was investigated by an FE-SEM (Merlin, Carl Zeiss AG, Oberkochen, Germany) with an Inlens SE detector at an accelerating voltage of 2 kV.

To analyze the thermal properties and the degree of crystallinity Χc [%] of the polyamides and their composites a differential scanning calorimeter (Q2000-TA, UB TA Instruments, Eschborn, Germany) was utilized using the following equation:

X_c_ = ΔH_m_/w·ΔH_m_^0^ (1)

where w is the weight fraction of PA in the composites, ΔH_m_ is the measured heat of fusion, and ΔH_m_^0^ represents the melting enthalpy of theoretical 100% crystalline PA 6.10 and PA 4.6 of 254 J/g and 210 J/g, respectively [26].

Furthermore, the coefficient of thermal expansion (CTE) α, was measured with a dilatometer (DIL 402 Expedis Supreme, Erich Netzsch GmbH & Co. Holding KG, Selb, Germany). 

**Figure 2:**
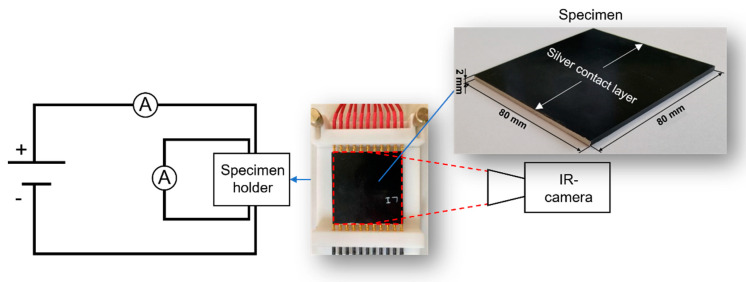
Measuring setup to analyze the self-heating properties and changes of electrical resistance of the specimens during an applied voltage of 24 V. The average surface temperature and the temperature of the hottest area (hotspot) over the entire sample surface of the nanocomposites were tracked.

The self-heating properties of the specimens were analyzed under an applied voltage of 24 V for 4 min with a voltage source (ES 030-10, Delta Elektronika B.V., Zierikzee, the Netherlands). In view of the planned use of the materials in the automotive industry for passenger cars/commercial vehicles, a maximum voltage of 24 V was tested. Changes in electrical resistance and surface temperature were tracked with a digital multimeter (Voltcraft VC 830, Conrad Electronic SE, Hirschau, Germany) and thermography camera (Thermol-Mager TIM 160 (48°), Micro-Epsilon Messtechnik GmbH & Co. KG, Ortenburg, Germany), respectively (Figure 2).

Further thermo-electrical characterizations, such as aging effects and changes of the electrical resistance of the specimens without self-heating, during temperature cycles from −24 °C to 150 °C and −24 °C to 193 °C, with heating/cooling rates of 1 K/min, were performed in a climatic chamber (VCS 7060-5, Weiss Technik GmbH, Reiskirchen, Germany). 

## 3. Results and Discussion

### 3.1. General Characterization

#### 3.1.1. Specific Surface Area

**Figure 3:**
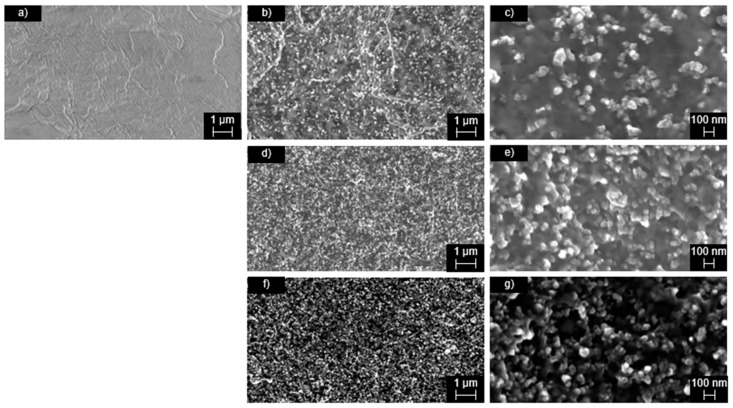
SEM images of neat PA 6.10 (**a**), 20 wt.% CB I (**b**,**c**), 20 wt.% CB II (**d**,**e**) and 20 wt.% CB III (**f**,**g**). (**a**,**b**,**d**,**f**) are shown with a magnification of 10.0 k, while (**c**,**e**,**g**) shown with a magnification of 50.0 k.

The specific surface areas of CB with 67 m^2^/g (CB I), 793 m^2^/g (CB II), and 1033 m^2^/g (CB III) showed low deviations from the data given by the suppliers. It is widely known that the dispersion of conductive fillers in the polymer matrix and the resulting robustness of the filler network is one of the critical parameters that dominate the thermal and electrical properties of the composite. Therefore, the microstructure, depending on the specific surface area of CB was visualized with SEM images (Figure 3). At low specific surface area (Figure 3c), less connected CB particles are exhibited, which do not ensure a three-dimensional filler network. Composites incorporated with high specific surface area CB (Figure 3e,g) provided continuous filler networks, with less free volume between the CB particles.

#### 3.1.2. DSC and Tg

**Figure 4:**
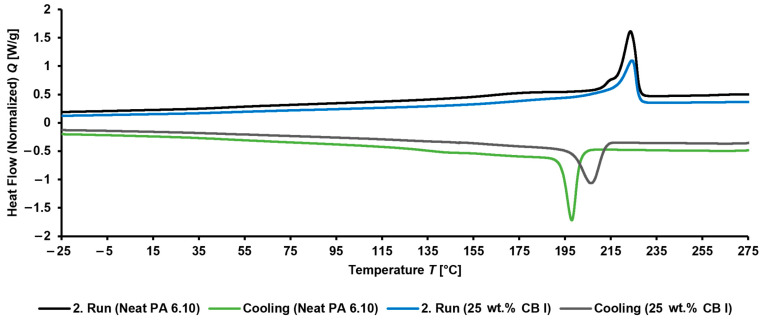
DSC measurement (2. run) of neat PA 6.10 and PA 6.10 with 25 wt.% CB I.

DSC measurements (Table A1) showed differences between neat polyamides and their composites (Figure 4) but no or even low deviations within the composites depending on the incorporated CB type or CB content. The crystallization behavior changed to higher temperatures of the crystallization onset and exothermic peak, which indicates earlier nucleation due to CB particles. Neat PA 6.10 showed a degree of crystallinity Χc of about 39%, whereby the values of its composites were between 37% and 42%. For PA 4.6 and its composites, crystallinities of 45% and 46% to 50% were analyzed, respectively. A similar observation of increased crystallinity was made for CB in PA 4.6 [27], multiwalled carbon nanotubes in polyamide 12 [28], or multiwalled carbon nanotubes in polypropylene [29]. All of them somehow addressed the nucleation-promoting effect of the fillers.

#### 3.1.3. Specific Resistance at Room Temperature

**Figure 5:**
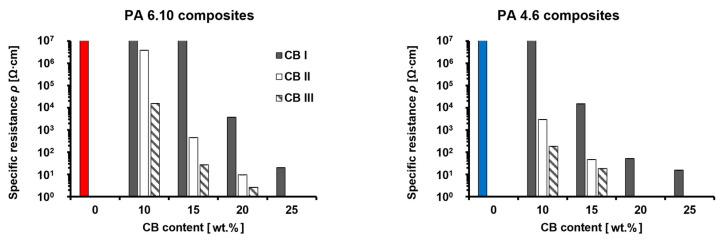
Shows the average specific resistance ρ of five specimens. PA 6.10/CB (left columns) and PA 4.6/CB (right columns) where neat PA 6.10 and neat PA 4.6 are represented by red and blue colors, respectively. The values of neat PA 6.10 and PA 4.6 are 6.5·10^12^ Ω·cm and 10^15^ Ω·cm, respectively.

It was found that the specific resistance of the specimens at room temperature decreased with increasing contents and specific surface area of CB, as well as higher polymer crystallinity (Figure 5). 

Because the CB content increased, there were more contact chances of CB particles, and more conductive paths were formed. The electrical behavior, depending on the filler content [30,31] or specific surface area of CB [32,33], has already been demonstrated in other studies. 

Using the high crystalline PA 4.6 provided further lower specific resistances of the composites compared to lower crystalline PA 6.10. Especially at 10 wt.% CB, the composites showed significantly lower resistances. This characteristic can be attributed to the behavior of semi-crystalline polymers. It has been verified that fillers predominantly tend to be located in the amorphous phase of semi-crystalline polymers. Increasing the overall crystallinity, crystallite size, and level of crystallite perfectness reduces the ratio of the amorphous to the crystalline phase. This leads to less free volume within the filler-containing, amorphous phase and improves the contact probability between the particles. Investigations to support this phenomenon have been carried out for MWCNTs in high and low crystalline polypropylene (PP) that showed a lower percolation threshold for high crystalline PP [34]. A similar research proposed tempering effects on CB containing PA 4.6, where the crystallinity of the composites increased, and the electrical resistance of the composites was reduced [27]. However, during the manufacturing of composite materials based on highly crystalline polymers such as polyamide 4.6 and carbon black, significant embrittlement must be accepted. This was found to be the case during compounding. Especially a carbon black content of 20 wt.% was not feasible, as the filaments exiting the compounder broke, and it was not possible to run a stable process.

Based on the results of this work, it can be established that the specific resistance can effectively be decreased by CB with a high specific surface area and even more by using high crystalline polyamide.

#### 3.1.4. Thermo-Electrical Properties

**Figure 6:**
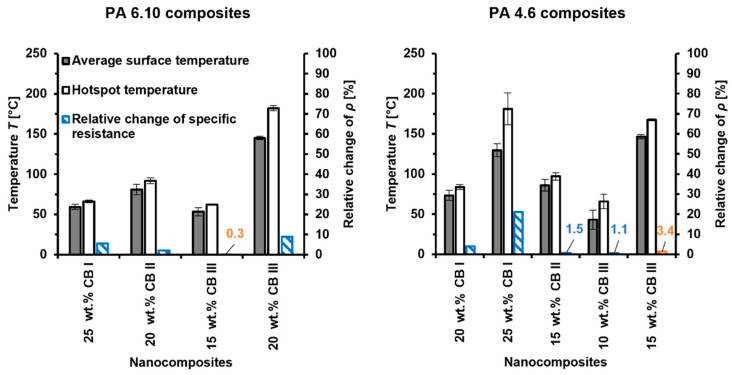
The average surface temperature and the temperature of the hottest area (hotspot) over the entire sample surface of the nanocomposites at an applied voltage of 24 V for 4 min. The relative change of resistance highlighted in blue and orange, represent a PTC-Effect and NTC-Effect, respectively.

Nanocomposites that exhibited specific resistances lower than 4 × 10^1^ Ω·cm showed self-heating properties (Figure 6). As with the specific resistance, high filler contents, specific surface area, and polymer crystallinity are shown to be conducive to the appearance of self-heating, which corresponds to the high robustness of the conductive CB network.

The maximum achievable surface temperature was raised for higher CB contents, such as for CB III in PA 6.10 and PA 4.6, or CB I in PA 4.6. This filler content behavior has also been demonstrated for graphene in epoxy composites [35]. High polymer crystallinity promoted self-heating further, which can be seen for PA-4.6-based composites, where lower CB contents were necessary to achieve high surface temperatures. Even 15 wt.% CB II in PA 4.6 showed a surface temperature of 86 °C, while 15 wt.% CB II in PA 6.10 showed no self-heating and 20 wt.% CB II heated up to 81 °C. It has been clarified that composites with decreasing specific resistance achieved higher material temperatures during self-heating [35]. For the reason that high polymer crystallinity leads to low specific resistances, it is obvious that this also results in a better self-heating performance. This correlation between low specific resistance and high self-heating performance of composites can be imposed on the specific surface area of CB as well. 

It was found that CB with a higher specific surface area accomplishes a better self-heating performance than CB with a low specific surface area. This can be seen for 20 wt.% CB II and CB III in PA 6.10, where surface temperatures of 81 °C and 145 °C were achieved. Similar behavior was found for 15 wt.% CB II and CB III in PA 4.6. 

Compared to other heating materials (Table 3), a higher voltage was tested in this study and higher material temperatures were achieved. However, the PTC behavior was not a part of this investigation, since only the heating performance was focused on. 

**Figure 7:**
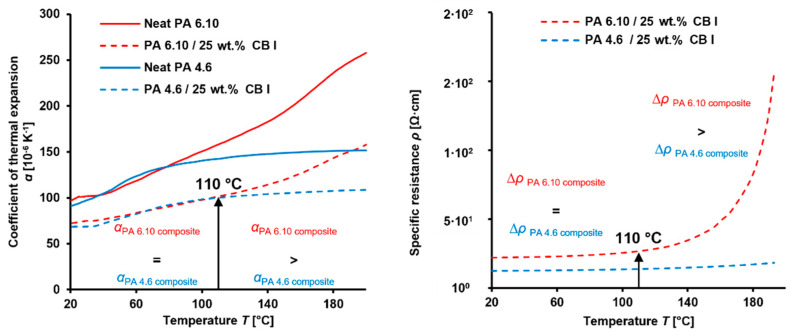
Coefficients of thermal expansion of neat PA 6.10 and PA 4.6 and their composites containing 25 wt.% CB I (**left** graph) and their changes of specific resistance within the same temperature range (**right** graph).

With the view of the PTC-Effect, the specific resistance of composites showed the well-known strong temperature dependency, which correlates with the thermal expansion of the polymer matrix [41]. To verify the influence of the thermal expansion of the polymer within the same temperature range, composites with 25 wt.% CB I in PA 6.10 and PA 4.6 were analyzed (Figure 7). It can be highlighted that neat PA 6.10 and PA 4.6 provided comparable coefficients of thermal expansion (CTE) up to ~80 °C. Above this temperature, PA 6.10 showed a higher CTE than PA 4.6. The same behavior can be seen for their composites but with the difference that their CTEs were comparable up to ~110 °C. Their changes in specific resistance correlate with their CTEs. PA 6.10 with 25 wt.% CB I showed a significant PTC-Effect starting around 110 °C compared to the PA-4.6-based composite where no clear PTC-Effect occurred. Interestingly, this PTC-Effect was reversed during their self-heating experiments, where the PTC-Effect was predominating for the PA 4.6 composite. This can be attributed to its higher temperature of about 147 °C compared to the PA 6.10 composite which reached 59 °C.

Furthermore, composites containing CB with a high specific surface area seem to reduce the PTC behavior. Especially for PA 4.6 composites, lower PTC Effects appeared for CB II and CB III compared to CB I. This phenomenon of specific surface area and PTC behavior have been demonstrated for CB in HDPE, but with one difference. The PTC performance has not been analyzed during self-heating [42].

Even a decrease in specific resistance has been analyzed, which could be attributed to the NTC characteristic of neat CB. 

Due to the different temperatures of the composites, there is an obvious need to carry out further analysis to clarify the PTC behavior of the composites. Therefore, they were stored in a climatic chamber at different temperature cycles to verify the PTC-Effects at comparable material temperatures. Due to the high water absorption, especially for PA 4.6 of approx. 3.5%, the moisture was removed during the first temperature cycle via the extraction system and the climatic chamber was subsequently sealed airtight.

**Figure 8:**
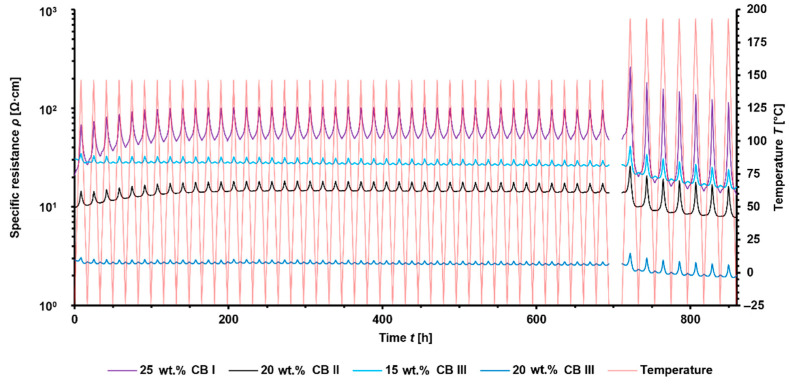
Thermo-electric performance of PA-6.10-based composites during temperature cycles from −24 °C to 150 °C and −24 °C to 193 °C.

Slight increases in the overall specific resistances during the first temperature cycles and stable thermo-electrical performance afterward were observed (Figure 8). However, during the temperature cycles up to 193 °C, the overall specific resistance decreased, which can be attributed to the re-aggregation of CB. This result is comparable to annealing experiments [43] that seem to improve its robustness and connectivity within filler networks. 

Significant expanded PTC-Effects with increasing temperatures have been observed, which has also been proven during self-heating of the composites. The highest PTC-Effect could be seen for the CB-I-containing composite. Interestingly the PTC-Effect of 25 wt.% CB I in PA 6.10 showed a higher PTC-Effect than 25 wt.% CB I in PA 4.6 compared to the PTC-Effect during the self-heating trials. As mentioned before, this is attributed to the temperature being higher than 25 wt.% CB I in which PA 6.10 could not be reached due to self-heating.

**Figure 9:**
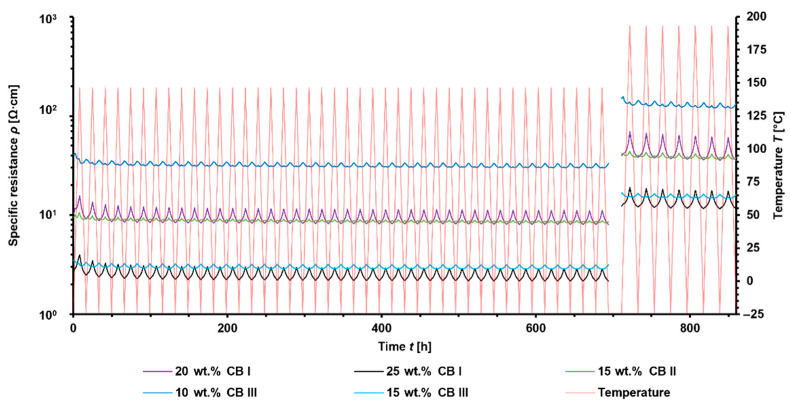
Thermo-electric performance of PA 4.6 based composites during temperature cycles from −24 °C to 150 °C and −24 °C to 193 °C.

PA-4.6-based specimens showed the same temperatures, depending on the increase of specific resistance during the temperature cycles and the highest PTC-Effect for CB-I-containing composites (Figure 9). Furthermore, the low thermal expansion of PA 4.6 is limiting the PTC-Effect compared to PA 6.10, which can be seen for their composites with 25 wt.% CB I or 15 wt.% CB III. 

Interestingly, PA-4.6-based composites showed a significant jump in the overall specific resistance from temperature cycles within a temperature range from −24 °C to 150 °C to temperature cycles from −24 °C to 193 °C. PA-6.10-based composites did not show such behaviors. It has already been demonstrated that PA 4.6 can induce a significant change in the degree of crystallization by annealing [27]. From the results obtained in this work, it is quite conceivable that the heating and cooling cycles could have changed the crystallization behavior to the detriment of the carbon black network, or that polyamide 4.6 is more susceptible to aging processes, which would explain the higher overall resistance. However, further analysis must be performed to support this assumption.

## 4. Conclusions

In this study, different types of carbon black with well-defined specific surface areas, were incorporated into PA 6.10 or PA 4.6 with different degrees of crystallinity. For the first time, the self-heating and self-regulating performance of these material compositions were investigated to provide a facile route to manufacture high-performance PTC-Heaters.

It was shown that sufficiently low specific resistances at room temperature and high self-heating properties can be achieved by using a highly crystalline polymer with CB of low specific surface area or less crystalline polymers with CB of high specific surface area.

The achievable temperature of the composite materials of up to 147 °C shows a considerable performance spectrum and can be individually achieved by the variable material composition disclosed in this study. 

Regarding the material costs and the achievable heating performance of the PTC heaters, it has become clear that the incorporation of low-cost carbon blacks with a low specific surface area, or only small amounts of expensive carbon black with a high specific surface area, into a highly crystalline polyamide are effective ways to provide industrially interesting heating solutions. 

Considering the self-regulating performance, high PTC-Effects of ~53% were shown, which is remarkable since the material temperature was well below the melting temperature of the polymer. Small differences in the PTC behaviors were revealed during self-heating and the passive measurements in the climatic chamber. For this reason, further studies need to be conducted. However, it is evident, that temperature differences within the specimens during self-heating, which can be seen in the average surface and hotspot temperature, could be one reason.

The following effects are the notable observations from the current material study:

- The most effective way to reduce the specific resistance and provide significant self-heating properties of the specimens is fulfilled by the incorporation of high amounts of CB with a high specific surface area into polymers with a high degree of crystallinity.
- The highest PTC-Effects were shown for composites incorporated with CB of low specific surface area.
- The occurrence of a PTC-Effect can be adjusted by the filler content and their extent depends on the BET of CB and the temperature of the specimens.
- Good reproducibility of the thermo-electrical performance has been achieved

Approaches to further improve the performance of the composites are also available. By adjusting the process parameters during compounding, higher carbon black contents of the composite materials could be achieved. As a result, the lower electrical resistances of the materials should provide higher heating performance. 

By increasing the applied voltage to the samples, higher temperatures should also be achievable. However, this carries the risk of local material degradation due to the hotspots already observed in this study. However, appropriate heat dissipation could address this issue, but this needs to be investigated in further studies. 

This work shows that self-heating nanocomposites can be well adjusted in their heating performance and PTC behavior to provide a large variety of high-performance heating solutions. 

## Figures and Tables

**Table 1:** Injection volume and speed of PA 6.10 and PA 4.6* composites. Values marked with * or without represent the PA-4.6-based composites and PA-6.10-based composites, respectively.

PA 6.10 and PA 4.6* Based Composites				
Injection volume [cm^3^]	32/38 *	11/11 *	10/10 *	0/0 *
Injection speed [cm^3^/s]	100/160 *	100/160 *	80/80 *	80/80 *
Neat PA 4.6				
Injection volume [cm^3^]	36 *	12 *	10 *	0 *
Injection speed [cm^3^/s]	100 *	100 *	80 *	80 *
Neat PA 4.6/15 wt.% CB II				
Injection volume [cm^3^]	37 *	10 *	9 *	0 *
Injection speed [cm^3^/s]	160 *	160 *	100 *	100 *

**Table 2:** Injection molding temperatures of PA 6.10 and PA 4.6* composites. Values marked with * or without represent the PA-4.6-based composites and PA-6.10-based composites, respectively.

PA 6.10 and PA 4.6* Based Composites		PA 4.6/15 wt.% CB II
Cylinder 1	270/315 *	315 *
Cylinder 2	260/305 *	305 *
Cylinder 3	250/295 *	295 *
Nozzle	270/350 *	335 *
Mold (Ejector side)	133/128 *	128 *
Mold (Nozzle side)	120/109 *	109 *

**Table 3:** Parameters and performance of differently composed heating materials.

Materials	Voltage *U* [V]	Temperature *T* [°C]	Source
PDMS/carbon nanotubes	7	~140	[36]
HDPE/carbon black	15	~100	[37]
LLDPE/carbon fiber	13	~68	[38]
PET fabrics/graphene oxide	14	~100	[39]
TPU/carbon black	20	~90	[40]

## Data Availability

Not applicable.

## Appendix A

**Table A1:** DSC measurements of PA 6.10, PA 4.6, and their composites.

Compound	*T*g [°C]	*T*m [°C]	Δ*H*m [J/g]	*T*c (Onset) [°C]	*T*c (Peak) [°C]	Xc [%]
PA 6.10	48	224	98	202	198	39
PA 6.10 + 10 wt.% CB I	48	224	92	211	205	41
PA 6.10 + 15 wt.% CB I	48	223	89	211	206	42
PA 6.10 + 20 wt.% CB I	48	223	86	213	207	42
PA 6.10 + 25 wt.% CB I	48	224	79	213	207	42
PA 6.10 + 10 wt.% CB II	46	223	89	208	203	39
PA 6.10 + 15 wt.% CB II	46	224	84	208	202	39
PA 6.10 + 20 wt.% CB II	45	225	79	208	202	39
PA 6.10 + 10 wt.% CB III	46	225	86	208	202	38
PA 6.10 + 15 wt.% CB III	45	224	83	209	202	39
PA 6.10 + 20 wt.% CB III	47	223	74	209	203	37
PA 4.6	76	289	95	261	257	45
PA 4.6 + 10 wt.% CB I	75	292	90	275	269	48
PA 4.6 + 15 wt.% CB I	77	292	89	276	270	50
PA 4.6 + 20 wt.% CB I	77	292	81	277	271	48
PA 4.6 + 25 wt.% CB I	76	292	79	277	270	50
PA 4.6 + 10 wt.% CB II	76	291	92	275	269	49
PA 4.6 + 15 wt.% CB II	76	292	88	275	269	50
PA 4.6 + 10 wt.% CB III	75	291	90	275	269	48
